# Mechanisms of Maternal Diet-Induced Obesity Affecting the Offspring Brain and Development of Affective Disorders

**DOI:** 10.3390/metabo13030455

**Published:** 2023-03-20

**Authors:** Daniel E. Radford-Smith, Daniel C. Anthony

**Affiliations:** 1 Department of Psychiatry, University of Oxford, Warneford Hospital, Warneford Lane, Oxford OX37JX, UK; 2 Department of Chemistry, University of Oxford, Mansfield Road, Oxford OX13TA, UK; 3 Department of Pharmacology, University of Oxford, Mansfield Road, Oxford OX13QT, UK

**Keywords:** gut microbiota, microbial metabolites, depression, metabolic disease, obesity, maternal nutrition

## Abstract

Depression and metabolic disease are common disorders that share a bidirectional relationship and continue to increase in prevalence. Maternal diet and maternal behaviour both profoundly influence the developmental trajectory of offspring during the perinatal period. At an epidemiological level, both maternal depression and obesity during pregnancy have been shown to increase the risk of neuropsychiatric disease in the subsequent generation. Considerable progress has been made to understand the mechanisms by which maternal obesity disrupts the developing offspring gut–brain axis, priming offspring for the development of affective disorders. This review outlines such mechanisms in detail, including altered maternal care, the maternal microbiome, inflammation, breast milk composition, and maternal and placental metabolites. Subsequently, offspring may be prone to developing gut–brain interaction disorders with concomitant changes to brain energy metabolism, neurotransmission, and behaviour, alongside gut dysbiosis. The gut microbiome may act as a key modifiable, and therefore treatable, feature of the relationship between maternal obesity and the offspring brain function. Further studies examining the relationship between maternal nutrition, the maternal microbiome and metabolites, and offspring neurodevelopment are warranted to identify novel therapeutic targets.

## 1. Introduction

Obesity is a common yet highly preventable metabolic disorder prevalent worldwide [1]. An audit led by the Royal College of Obstetricians and Gynaecologists in 2017 [2] discovered that a quarter of pregnant women in the UK are obese, with a similar prevalence across continental Europe [3]. Moreover, fewer than half of women in the UK have a pre-pregnancy body mass index (BMI) within the normal range [2]. Excessive gestational weight gain (GWG) is also a relatively common occurrence during pregnancy in the UK and other Western countries, including the USA and Australia [4]. Entering pregnancy with an elevated BMI increases the likelihood of excessive GWG, as well as the risk of retaining weight postpartum [5]. Independent of pre-pregnancy BMI, 40% of women in Western countries gain excessive weight during pregnancy [4]. It has also been shown that the majority of pregnant women gain excessive weight due to the consumption of a diet high in fat [6].

**Figure 1:**
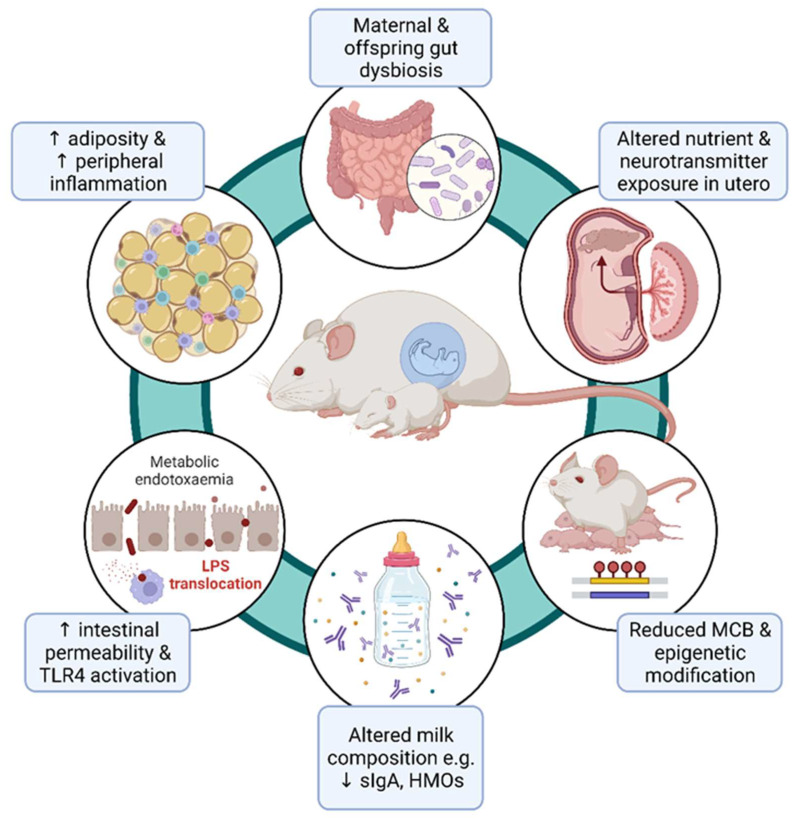
Summary of the putative mechanisms by which maternal obesity is thought to influence the course of offspring neurodevelopment. MCB, Maternal care behaviour; sIgA, secretory immunoglobulin A; HMOs, human milk oligosaccharides; TLR4, toll-like receptor 4.

While the consequences of maternal obesity on offspring metabolism, obesity, and cardiovascular health have been widely reported [7,8], only more recently are thorough investigations being made into how maternal diet and obesity affect maternal and offspring behaviour, including the transmission of psychiatric disease risk through poor maternal health during gestation and the early life of the offspring. These potential mechanisms have been summarised in Figure 1. It is critical to have a mechanistic understanding of how the maternal uterine and lactational environment may mediate offspring neurodevelopmental morbidity, because pregnancy may represent an important window for targeted intervention to ameliorate foetal and offspring risk [9].

Seminal studies in rodent [10] and nonhuman primate (NHP) [11] offspring first observed the deleterious effects of maternal peripartum high-fat diet feeding on the offspring brain and behaviour (Table 1). In rats, it was shown that the expression of CD11b, a marker of macrophages and CNS-resident microglia, and TLR4 was significantly upregulated in the juvenile offspring of obese dams fed a diet high in saturated fat [10]. TLR4 activation is required for robust pro-inflammatory responses to bacterial endotoxin, but it also mediates central inflammatory signalling and alters brain metabolism. In addition, the basal level of IL-1β protein was significantly elevated in the hippocampus and periphery (liver) of both juvenile and adult offspring of high fat diet (HFD)-fed dams compared to offspring of controls. These inflammatory changes in HFD-exposed offspring were associated with an increased anxiety in the elevated plus maze. Moreover, Sullivan et al. (2010) linked changes in maternal HFD (mHFD) consumption during gestation with an increased anxiety-like behaviour in juvenile female NHP offspring [11]. This was also accompanied by perturbations to the serotonergic system, including decreased cerebrospinal fluid levels of serotonin. These studies paved the way for future animal models investigating the underlying mechanisms behind diet-induced obesity during gestation and nursing and altered neurobiology and behaviour in the offspring (Table 1).

## 2. Clinical Studies

Meanwhile, clinical studies, largely epidemiological in nature, have highlighted the translational relevance for this field of experimental research (Table 2). Some of the first association studies in humans investigated the link between low birthweight—a complication of pre-term birth with significantly greater prevalence in maternal obesity [22]—and behavioural problems in children. Initially, it was shown that a very low birth weight contributed to education difficulties and hyperactive behaviour in children [23,24], independent of sociodemographic factors. Attentional problems were the focus of early studies in low-birthweight offspring [23,25,26,27,28,29,30,31,32,33,34]. Following this, Rodriguez et al. (2008) made the direct association between maternal obesity and attention deficit hyperactivity disorder (ADHD), independent of birthweight [35].

A 2010 study showed that, in addition to inattentiveness, pre-pregnancy obesity and overweightness predicted difficulties with emotional intensity and regulation in children [36]. Studies over the subsequent decade affirmed the relationship between an elevated pre-pregnancy BMI and internalising and externalising behaviours [37,38,39,40]. Here, internalising behaviours correspond to affective disorders, whereby problems of an individual are internalised, manifesting in symptoms of depression, social withdrawal (as opposed to antisocial behaviour), and anxiety. Other, related behavioural abnormalities, characterised by high levels of negative effects, include obsessive-compulsive disorder, dissociative disorders, and eating disorders. In externalising disorders, on the other hand, emotions and cognitions are manifested as maladaptive, antisocial behaviours towards the external environment. ADHD is a relatively common externalising disorder. Despite their opposing behavioural phenotypes, these categories often co-occur in a household or individual, and appear to have a shared aetiology [41]. Throughout childhood and adolescence, both internalising and externalising behavioural problems are more likely to occur after gestational and early-life exposure to maternal obesity [38]. An analysis of the same pregnancy cohort followed up children prospectively between the ages of 5 and 17 and found a higher risk of affective disorder in those exposed to an elevated pre-pregnancy BMI [37]. Importantly, these and more recent studies with consistent findings adjust for potential causative and confounding factors: weight gain during pregnancy, birthweight, and the presence of maternal psychiatric disorders were common adjustments across these studies [39,40,42] (Table 2). More rigorous considerations were made in the most recent investigations, including the maternal age, delivery method, country of birth, socioeconomic status, marital status, smoking status, and presence of a systemic inflammatory disease; for example, Crohn’s disease [42]. The continued clinical relevance of this transgenerational relationship between obesity and behaviour, coupled with highly consistent findings in humans, highlights the need to uncover specific mechanisms of transmission and accompanying therapeutics. While the relationship is undoubtedly complex, with multiple contributing factors [9], establishing the key pathways involved will pave the way for more targeted prevention and treatment during gestation and early-life offspring.

**Table 2:** Overview of clinical studies demonstrating an association between maternal obesity and altered behavioural outcomes in children independent of other covariates.

Maternal Risk Factor	Child Outcome (Age at Follow-Up)	Study Design and Sample Size	Covariates Investigated	Author, Year
↑ Pre-pregnancy BMI and/or gestational weight gain	↑ teacher-rated ADHD symptomology (7–8 years or 10–12 years)	Cohort study, N = 12,556	Maternal age, social adversity, maternal smoking, gestation length, birth weight, infant sex, maternal education, family structure	Rodriguez et al., 2008 [35]
	↑ teacher-rated ADHD symptomology (5 years)	Cohort study, N = 1714		Rodriguez, 2010 [36]
Pre-pregnancy obesity	↑ Affective behavioural problems as determined by primary caregiver (5, 8, 10, 14, 17 years)	Longitudinal cohort study, N = 1754	Maternal age, maternal education, household income, maternal smoking, alcohol consumption, family structure, maternal life stress, gestation length, gestational diabetes, duration of breastfeeding, birth weight	Robinson et al., 2012 [37]
↑ Pre-pregnancy BMI	↑ Internalising and externalizing problems in childhood and adolescence (5, 8, 10, 14, 17 years)	Longitudinal cohort study, N = 2868	Maternal psychopathology, paternal psychopathology, maternal age, alcohol intake, smoking, socioeconomic status, education, marital status, maternal life stress, maternal diabetes	Van Lieshout et al., 2013 [38]
Pre-pregnancy obesity	↓Child psychosocial development at age 6↑ likelihood of prior ADD/ADHD diagnosis, speech/language therapy, psychological or special needs services (6 years)	Cohort study, N = 1542	Maternal age, ethnicity, marital status, education, parity, household income, maternal smoking, infant sex, infant current weight, child enrichment activities (e.g., reading to child >3 times per week), breastfeeding duration, postpartum depression, birth weight	Jo et al., 2015 [39]
↑ Pre-pregnancy BMI, pre-pregnancy and gestational diabetes	↑ risk of neurodevelopmental, ADD/ADHD, psychotic, mood, stress-related disorders in offspring (up to 11 years, median 5.5 years)	Population study, N = 649,043	Infant birth year, infant sex, perinatal problems, gestation length, parity, mode of delivery, maternal age, family structure, ethnicity, maternal smoking, maternal inflammatory comorbidities	Kong et al., 2018 [40]
			Infant birth year, infant sex, perinatal problems, gestation length, parity, mode of delivery, maternal age, family structure, ethnicity, maternal smoking, maternal inflammatory comorbidities, maternal psychopathology	Kong et al., 2020 [42]

AD(H)D, attention deficit hyperactivity disorder; BMI, body mass index.

## 3. Maternal Factors Influencing Offspring Neurodevelopment and Behaviour

### 3.1. Maternal Care Behaviour

It is generally accepted that the importance of the relationship between the mother and infant transcends nutritional need. This was demonstrated in animals in early ethological studies [43,44], which initially demonstrated the impact of contact and comfort on primate development independent of nutritional support. Infant primates raised in the absence of maternal care developed severe social deficits while developing strong emotional attachment to inanimate objects that provided any source of comfort. While infants during the early postnatal period are typically hyporesponsive to stress compared to other life stages, maternal care (and neglect) remains a strong influence over neurodevelopment [45].

Clinical studies have revealed important associations between childhood maltreatment and altered neurobiology and behaviour. Childhood neglect, in its various forms, is associated with an increased risk of behavioural problems and psychiatric illness. Longitudinal studies are important for clinical studies on the effects of maternal care. A large longitudinal study in 2017 showed that poor maternal care provides context for a link between depression and elevated BMI in adult women [46]. This and other clinical studies indicate that female offspring may be more at risk of developing psychopathologies as a consequence of poor maternal care. Another study showed that female adolescents of mothers with major depressive disorder (MDD) have a significantly greater likelihood of developing a psychiatric disorder [47]. Exposure to maternal neglect in childhood is also a risk factor for attempted suicide, depression and anxiety, and other psychiatric disorders [48]. A comprehensive systematic review of clinical studies on different forms of childhood maltreatment found neglect to have one of the highest associations with the development of depressive disorders in later life [49]. The environmental modulation of development during early life likely serves to allow for the adaptation of subsequent generations to novel environments that may differ to those of previous generations. During the perinatal period, the developing brain is particularly susceptible to environmental insult, including neglect. Prevailing theory suggests that exposure to early stress through a lack of maternal (and paternal) care programs enhanced the responsiveness to stress [50,51]. While this may be viewed as a neurodevelopmental adaptation to a perceived stressful environment, it is almost certainly detrimental in the present environment. Understanding the neurobiological sequelae of exposure to poor maternal care is therefore an important feature of both clinical and preclinical neuroscience and behavioural research.

The abnormal development of specific brain regions has been implicated in maternal neglect. Both the hippocampus and prefrontal cortex (PFC) undergo protracted postnatal development and neurogenesis prior to myelination and synaptic pruning, with the hippocampus continuing to develop during childhood and the PFC developing into the third decade of life [52,53]. Both regions have a high density of glucocorticoid receptors (GRs) [54,55]. Physiologically, these GRs serve to maintain homeostasis by inhibiting further hypothalamic pituitary adrenal (HPA) axis activity during the stress response. They are also thought to be important to neuronal function and survival through the regulation of brain-derived neurotrophic factor (BDNF), a key protein involved in the regulation of brain synaptic plasticity [56].

In rodents, maternal neglect has been shown to affect brain development and behaviour via overactivation of the HPA axis. Temporary intervals of maternal separation from pups activate the HPA axis, increasing the levels of circulating adrenocorticotropic hormone (ACTH) and glucocorticoids [57]. Prolonged separation reduces corticotropin releasing hormone (CRH) binding sites in the hypothalamus [58], possibly due to negative feedback via the sustained elevation of circulating stress hormones. Lower levels of maternal care, specifically the reduced licking and grooming of pups, correlates with reduced hippocampal GR mRNA levels and increased CRH mRNA in the hypothalamus [59]. Moreover, chronic corticosterone administration has been shown to hinder neurogenesis in the hippocampus in rodent models in a number of independent studies [60]. Brain transcriptomic analysis has subsequently revealed that maternal care behaviour regulates the expression of >900 genes in the hippocampus [61]. Finally, it has been shown that low levels of maternal licking and grooming behaviour lead to persistent anxiety-like behaviours in the adult (3-month old) offspring [61,62].

Poor maternal care has also been shown to increase anxiety behaviours in NHP offspring, which may be mediated by perturbations to tryptophan metabolism via increased glucocorticoid levels. Macaques exposed to maternal neglect displayed elevated anxiety-like behaviours (increased scratching behaviour) between 12 and 24 months of age, which correlated with reduced levels of 5-hydroxyindoleacetic acid (5-HIAA) in the cerebrospinal fluid (CSF) [63]. 5-HIAA is a major metabolite of serotonin and may indicate reduced brain serotonin synthesis. While glucocorticoids were not measured, other studies have shown social separation in early life to significantly increase plasma cortisol levels at the age of 6 months [64] and persistently elevate CRH in the CSF at the age of 2 years [65]. Glucocorticoids are known to induce the expression of tryptophan 2,3-dioxygenase, an enzyme that catalyses the first and rate-limiting step of tryptophan to kynurenine [66,67]. It is possible, therefore, that early life maternal separation increases circulating glucocorticoid levels, directing tryptophan metabolism towards kynurenine metabolism (in peripheral tissues or in the brain) and depleting brain tryptophan availability for serotonin synthesis. In parallel, early life stress has been shown to reduce the metabolism of kynurenine to kynurenic acid, a neuroprotective agent, and increase metabolism to quinolinic acid, a neurotoxic agent [68]. Given that serotonin levels peak during the perinatal period [69], it is unsurprising that perturbations to signalling during this sensitive period could produce long-lasting behavioural changes reminiscent of affective disorders [70]. Recent evidence [71] indicates an immediate impact of serotonergic signalling in response to maternal presence, whereby PFC activity during the early postnatal period is reduced during maternal absence as opposed to maternal presence. This was shown to be mediated by 5-HT2 receptors and is consistent with another study that demonstrated early life stress to increase the pre-mRNA editing (and reduce downstream activity) of the 5HT-2C receptor in adult mice [72].

The adverse effect of stress on neurodevelopment has been demonstrated retrospectively in humans by a reduced hippocampal volume in adults with a history of childhood trauma [73,74]. Women, but not men, who experienced low maternal care during early life have a reduced hippocampal volume compared to matched control individuals [75]. This points to the increased susceptibility of female offspring to poor maternal care. Similar findings have been identified in the PFC, whereby the grey matter volume in the prefrontal cortex has been shown to be reduced in children exposed to early life stress in both longitudinal studies and when evaluated retrospectively [76,77,78,79]. The total brain volume has also been shown to be reduced in children with a history of trauma, compared to typically developing children [76,80]. A reduced hippocampal volume is also a risk factor for future psychiatric disease [81]. Other studies have been more equivocal: De Bellis et al. (1999) found no effect of post-traumatic stress disorder on hippocampal volume in children or adolescents compared to control individuals [82]. Similarly, while Carrion et al. (2001) found that children (age 7–14) with a history of psychological trauma had a smaller total cerebral volume compared with control individuals, there was no difference in the relative volume of the hippocampus [80]. In summary, significant stress exposure prior to the complete development of the hippocampus and the PFC may lead to their precocious maturation at the expense of their full developmental potential [50,77].

Maternal depression has been demonstrated to be a modifying factor in the quality of maternal care behaviour, whereby depressive episodes are an obstacle to sensitive maternal care. Depressed mothers are less attentive and responsive to the needs of their offspring [83]. Substantial clinical evidence now exists to show that maternal depression affects the HPA axis of developing children. For example, children of mothers who exhibit depression show elevated morning cortisol levels at 6–10 years age [84]. In another study, elevated waking cortisol levels were identified in 13 year old children of mothers with postpartum depression [85]. In the same cohort, it was shown that the elevated cortisol levels in these children played a significant mediatory role in the development of depression at the age of 16 years [86]. Retrospective studies in humans have shown that both prenatal and postnatal maternal depression adversely affect cognitive and emotional development in offspring [87,88,89]. Furthermore, lower scores on parameters that measure parent–child relationships (i.e., reduced empathy and more emotional distance) significantly increase the risk of depression and anxiety in later life [90,91], increasing the risk of parental–offspring neglect and psychopathology between generations.

Preclinical studies investigating natural variations in maternal care (without human intervention, i.e., maternal separation from pups) in rats show that natural variations in the extent of the licking/grooming (LG) and arched-back nursing (ABN) of pups has a significant influence on neurodevelopment and behaviour. Offspring of dams engaging in higher levels of LG show an increased exploratory behaviour in an open field [92]. Another study showed that naturally occurring higher levels of LG and ABN increased the expression of NMDAR subunit and brain-derived neurotrophic factor (BDNF) mRNA, with an increased hippocampal synaptogenesis and improved spatial memory [59]. Changes in offspring gene expression and behaviour may be mediated by epigenetic mechanisms [91,93,94]. Altered DNA methylation due to natural variation in maternal care that leads to changes in the chromatin structure can result in the attenuation of hippocampal GR expression in pups exposed to less LG [93]. Interestingly, preclinical evidence also supports the inheritance of maternal care behaviours, such that female mice exposed to lower levels of maternal care also exhibit poor maternal care to subsequent generations via the epigenetic regulation of brain estrogen receptor alpha expression [94]. Association studies in humans have also been conducted that show that an abusive or neglectful parent is far more likely to have a history devoid of adequate parental care [95].

Few studies have investigated how the maternal diet or diet-induced obesity affects maternal care behaviours in rodents or the parenting style in clinical studies. A high-fat, high-protein diet in rats leads to increased ABN and reduced passive nursing (PN), as well as increased grooming behaviour [96]. This study is of limited validity as maternal care was observed from birth to weaning age (postnatal day 21), and only for 2 h each day per animal. Maternal care exhibits natural variation throughout this period, and a more intensive observational study in the first week of life might have been more meaningful [97]. Additionally, changes in maternal behaviour towards pups were not linked to any brain or behavioural changes in offspring. A more recent study, also using rats, looked at the effect of a high-fat diet (60% kcal from fat) in comparison to a control diet (10% kcal from fat) on maternal care behaviours from postnatal days 3 to 6 [98]. They found no differences in maternal care behaviour during the day (light period) over a 3 h observation for each day. During the dark period, ABN and total nursing was increased in dams exposed to a palatable high-fat diet, whereas time spent resting was reduced. No differences were observed between licking/grooming. This study also has several limitations; the rats were only started on the high-fat or control diets after the second day of gestation, and straight after arriving to the facility. The effect of transport stress may confound results of stress due to diet. Furthermore, weight gain in the mothers was not assessed, and it is unlikely that significant weight gain occurred during the feeding period. Short-term palatable high-fat diet feeding may reduce anxious behaviour rather than induce depressive-like behaviours and deficits in maternal care, particularly in the absence of obesity [99]. Moreover, evidence from another study suggests that an obese phenotype (significant weight gain) rather than high-fat diet exposure alone is required to adversely impact offspring neurodevelopment and behaviour [13]. A third study [14] using inbred mice showed that the dams of neonatal mice exposed to a high-fat diet in utero were less interactive than neonates exposed to a control diet. Using a cross-fostering design, this was demonstrated to be independent of the mothers’ diet, instead suggesting a role of offspring physiological changes that cue maternal interaction with pups. Importantly, females were fed the high-fat diet (45% of kilocalories from fat) for six weeks prior to mating. This is an interesting concept, suggesting that, even at this early stage, the relationship between the mother and offspring is instigated bidirectionally [100]. Another recent study of maternal care behaviour (MCB) in rats [15] used a high-fat diet (60% of kilocalories from fat) that started 3 weeks prior to mating. MCB was assessed between the first and sixth postnatal day for 3 h during the day and 3 h during the night (at spaced intervals throughout the day and night). In this study, high-fat dams were significantly heavier than their control counterparts. No differences between the control and high-fat diet groups were found. Conversely, our laboratory has shown that, after 9 weeks of HFD feeding, maternal mice are less attentive to their offspring during the first postnatal week of life (as measured by the time spent away from the nest) compared to maternal mice fed a control diet [12]. The average time spent nursing, licking, and grooming was not different between obese and lean dams. As nursing, licking, and grooming behaviours are thought to be the key actions that can influence offspring development [93,101], it is unlikely that the extra time spent away from the nest in obese dams would have adversely affected offspring development, as it appeared to only be at the expense of the average time spent nest building. While an adverse relationship between nest building and offspring developmental or behavioural outcomes has not been reported, nest building is a highly motivated behaviour during pregnancy [102] and its reduction in favour of inactivity may be indicative of anhedonia. Clearly, the preclinical evidence for an effect of high-fat-diet feeding on maternal care is mixed and warrants clarification in future studies. The length of high-fat-diet feeding may also affect MCB. It may be that maternal care deteriorates over longer levels of pre-pregnancy high-fat-diet feeding.

Preliminary evidence in human studies similarly indicates that the diet influences maternal behaviour, although confounding variables are more difficult to control. A systematic review found that a healthy diet prior to and during pregnancy reduces the risk of perinatal anxiety and depression [103]. Postpartum depression is also exacerbated by a poor diet [104]. On the other hand, a recent study conducted in the UK showed no evidence for the overall diet being linked to postpartum depressive symptomology in humans after adjusting for many potential confounders [105]. Thus, the evidence for the diet playing a significant modifying role on the maternal perinatal psychopathology is certainly mixed.

In summary, preclinical studies have begun to shed light on some of the key mechanisms mediating the effect of maternal neglect, a form of early life stress, on offspring neurodevelopment with consequences for behaviour. These concepts align with the theory of foetal programming, whereby exposure to significant amounts of neglect or other stressors during the early postnatal window directs brain development along a specific pathway best adapted to a stressful environment. In a benign environment, these behaviours are maladaptive and likely increase the risk of internalising and externalising behavioural disorders in later life. When investigating the effect of maternal-diet-induced obesity on maternal behaviours, the evidence is limited and mixed. Because of this and the established effect of maternal care on the offspring brain and behaviour, observed variations in maternal care should be reported in all studies investigating the gestational and early postnatal period, at least to eliminate possible confounding effects on other outcomes.

### 3.2. The Maternal Microbiome

The effect of maternal obesity on the offspring brain and behaviour may be mediated, in part, by changes to the maternal and offspring gut microbiome. The obesity status and consumption of a high-fat diet prior to and during pregnancy have been shown to affect the composition of the maternal and neonatal gut microbiome in humans [106,107,108] and rodents [109]. Moreover, maternal gut dysbiosis induced by diet [16] or antimicrobial treatment [110] has been shown to affect the offspring microbiome, as well as the offspring brain and behaviour.

While the absence of a placental microbiome has been confirmed in humans [111], preclinical studies in mice have nonetheless revealed a profound effect of the maternal microbiome on prenatal development through the transfer of microbial metabolites from the dam to the offspring. Microbial metabolites contribute to foetal nourishment, but are also crucial for the origins of immunity [112] and neurodevelopment [113,114]. Maternal chronic mild stress during the first week of gestation has been shown to affect the maternal vaginal microbiome composition through a decrease in *Lactobacillus* spp., markedly reducing the abundance of this taxa in offspring, with associated changes to the plasma metabolome affecting the energy balance and mitochondrial function [113]. The levels of brain amino acids glycine and threonine were also reduced in early-life (postnatal day [PND]2) male offspring but increased in early-life (PND2) female offspring as a consequence of maternal prenatal chronic mild stress. This is an interesting sex-specific observation and may account for differences in susceptibility to maternal stress in male and female offspring.

The specific absence of certain or all microbes during early life has been shown to affect behaviour in later life. For example, Sudo et al. (2004) showed that GF mice exhibit downregulated NMDA receptor subunit NR2A in response to acute restraint stress, which was reversed by the microbial reconstitution of Bifidobacteria (but not *Escherichia coli*) neonatally [115]. Stress-induced changes in brain gene expression were not reversible by gut microbial reconstitution in adulthood. This seminal study established the importance of the commensal microbiota in the normal conditioning of the HPA axis. An altered gut microbiota due to a poor maternal diet likely also has a pervasive effect on metabolites reaching the foetus, which may prime offspring for disease. Germ-free mice colonised with the gut microbiota of offspring born to obese dams have an increased intestinal permeability (determined by increased plasma fluorescein isothiocyanate dextran levels), hepatic inflammation (increased hepatic *TNF* mRNA and hepatic macrophage count), and susceptibility to non-alcoholic fatty liver disease (NAFLD), determined by a histological examination of liver sections for steatosis and the infiltration of inflammatory cells [116]. Clearly, maternal microbial changes that occur because of perinatal obesity affect the composition of vertically transmitted metabolites to offspring during foetal and neonatal development.

One of the first pieces of evidence linking a maternal high-fat diet to the offspring brain and behaviour via changes in the gut microbiota comes from a study by Buffington et al. (2016) [16]. In this study, female mice were fed a HFD (60% of kilocalories from fat) for 8 weeks prior to mating [16]. This is a more appropriate time period to achieve a mouse model of diet-induced obesity [117]. At parturition, litter sizes were significantly reduced compared to non-obese mothers who were maintained on a regular diet (13.4% kcal fat). Even though offspring mice were maintained on a ‘regular’ diet until adulthood, substantial differences in the gut microbiome were observed, mirroring some of the changes observed in the F0 maternal generation. These changes were replicated when offspring gut microbes were transplanted into germ-free mice. Alongside changes to the microbiome, the offspring of dams fed a HFD exhibited social interaction deficits, and, with this, reduced oxytocin levels and the potentiation of synaptic inputs to dopamine neurons in the ventral tegmental area (VTA) after the social interaction protocol. *Lactobacillus reuteri* was found to be the most reduced species in maternal HFD offspring compared to controls. A reconstitution of this species in maternal HFD offspring (4-week supplementation in drinking water post-weaning) reversed social and synaptic deficits, which was not the case for other depleted taxa. Heat-killed *L. reuteri* was unable to rescue social behaviour and had no effects on social-interaction-induced long-term potentiation in the VTA of maternal HFD offspring. While this study provided some of the first compelling evidence for the role of specific microbes in neuropsychiatric disorders influenced by maternal nutrition, it contradicts other evidence that functional metabolite production likely plays more of a role in mediating changes to the gut–brain axis, rather than specific taxa [118]. With this in mind, microbial metabolite production was not reported as a result of a maternal high-fat diet, and functional metabolite changes as a direct result of *L. reuteri* administration were not described, despite this species having been demonstrated as effective in promoting γ-Aminobutyric acid (GABA) and histamine production in vitro [119]. Maternal chronic variable stress exposure during the first week of gestation in mice has also been shown to reduce *Lactobacillus* spp. in the vaginal microbiome (early postpartum) and offspring gut [113]. Offspring exposed to early prenatal stress also had altered colon and plasma metabolite profiles, associated with energy and microbial metabolism. Finally, prenatal stress altered brain amino acid levels in offspring (postnatal day 2) in the hypothalamus and hippocampus. The effect of peripheral metabolic changes on the brain is particularly important during development as, relative to mature brain structures, certain brain regions are much more readily accessible to circulating factors [113]. Offspring behavioural outcomes were not explored in this experiment. It is currently unknown whether maternal obesity significantly alters the normal composition of the vaginal microbiome, and this could be a mechanism by which maternal obesity leads to vertically transmitted changes in the offspring gut microbiome, with implications for social behaviour [16].

The effect of diet-induced microbial changes, but not the high-fat diet itself, on offspring behaviour has also been explored [120]. This allows for the direct effect of the microbiota to be assessed, rather than the whole array of changes induced by a maternal high-fat diet, on offspring behaviour. Microbiota from male mice fed with a high-fat (60% kcal fat) diet for 3 months were transplanted into microbiota-depleted adult females. Obese-type microbiota did not induce weight gain or affect maternal care in dams, though female offspring were significantly heavier than control offspring at weaning and adulthood. In adult offspring, offspring males from dams with obese-type microbiota showed an increased anxiety relative to controls, though, in females, no changes were found. All offspring showed microbiome changes that differed in accordance with maternal treatment. This study provided further evidence that maternal microbiota may program aspects of offspring behaviour.

The intrinsic ability of microbes associated with obesity to alter brain function and behaviour has also been demonstrated [121]. Gut microbes from adult mice were depleted during a 2-week antibiotic protocol and recolonised with donor microbes from the caecal content of genetically identical mice fed an HFD (60% of kilocalories from fat) for 8 weeks. Compared to mice that received donor microbes from mice fed a control diet, microbes derived from a high-fat diet increased anxiety-like behaviours in the OFT, EPM, and marble burying test in the absence of any weight gain in recipient mice. Increased plasma endotoxin and TLR4 expression and brain macrophage marker IBA1 and TLR4 expression were indicative of increased peripheral and central inflammation, respectively. Given that microbiota may partially mediate neuropsychiatric consequences of obesity, it can reciprocally be suggested that the modulation of the microbiome in obese individuals, or individuals exposed to obesity with an altered microbiota composition, may be able to attenuate brain and behavioural deficits. The efficacy of such treatment may give rise to narrow therapeutic windows that correspond to important periods of neurodevelopment. For example, Buffington et al. (2016) [16] found that microbes donated from conventionally housed mice reversed social deficits in germ-free mice at 4 weeks old, but not at 8 weeks [16].

Diet-induced obesity also alters the innate immune response through changes in the gut microbiota composition. While it is well known that commensal microbes interact with local immune cell populations in secondary lymphoid organs such as the gut [122], the mechanism by which microbiota influence haematopoiesis at primary lymphoid organs—for example, the bone marrow—is less clear. Lee et al. (2019) demonstrated that a defined population of bone marrow resident macrophages are able to detect circulating cell-free bacterial DNA [123]. The detection of this DNA drives the production of cytokines such as TNF and IL-1B, which regulate the production of bone marrow haematopoietic stem cells (HSCs). While the complete absence of microbiota appears detrimental to primary immune cell development, modification to the microbiome by diet may also impact innate immune cell development. Luo et al. (2015) showed that an HFD altered the HSC niche in mice, with increased common myeloid as opposed to common lymphoid progenitors [124]. Increased myeloid progenitors may underly more potent inflammatory responses to infection in obese individuals [125] and increased peripheral and central *TLR4* expression [126]. This effect was found to be mediated, at least in part, by changes to the gut microbiota, as the depletion of gut microbes by oral antibiotics reversed changes to the HSC niche. Additionally, FMT from high-fat-diet mice to mice on a regular diet induced the same effect as the direct ingestion of the high-fat diet. By extension, high-fat-diet-induced changes to the microbiome could mediate depressive symptomology through immune dysfunction beginning in the HSC niche. Because innate immune memory appears to be transferable through the microbiota [127], and the neonatal microbiota is significantly shaped by the mother [128], an adverse combination of microbes and immunomodulatory compounds during early life may impact the brain and periphery of developing offspring.

There remains a scarcity of studies that have investigated how obesity-induced changes to the maternal microbiome impact offspring neurodevelopment and behaviour. Most studies have investigated the link between obesity, the gut microbiota, and mood disorders in a single generation [129]. Some evidence points to the deficiency of *Bifidobacteria* spp. or *Lactobacillus* spp. in obese subjects mediating reduced beneficial metabolites [130], though increased basal inflammation through increased circulating microbial endotoxins likely also plays a role [131]. Work in our laboratory has previously shown that maternal HFD-induced obesity reduces the abundance of *Bifidobacterium* spp. in maternal mice postpartum compared to maternal mice fed a control diet [12]. In the same study, maternal HFD feeding was found to negate the effect of probiotic supplementation on increasing faecal short-chain fatty acid (SCFA) levels, and increased maternal plasma cholesterol levels.

### 3.3. Maternal Inflammation

Perhaps the most striking evidence for the contributory role of maternal inflammation in programming offspring brain function and behaviour stems from the observation of maternal infection during pregnancy and the increased risk of neuropsychiatric disorders in the offspring. This has been investigated in preclinical models of maternal immune activation (MIA) and human epidemiological data. In humans, converging evidence from maternal autoimmune disorders, acute stress, and infection during pregnancy point to elevated maternal immune mediators in enhancing the risk of offspring psychopathology development. Moreover, viral infection during pregnancy can significantly alter normal neuronal development, as in the case of Zika virus infection [132]. In rodents, congenital Zika virus infection impacted learning and memory in adulthood, which presented with a reduced number of mature hippocampal neurons [133]. More generally, rodent MIA models demonstrate behaviours resembling autism spectrum disorders and schizophrenia, as well as anxiety-like behaviours [134]. The basal level of several neurotransmitters in different regions of the rodent brain have also been shown to be influenced by MIA and are thought to underly these persistent behavioural changes [135]. More recently, MIA has been associated with offspring depressive-like behaviour in preclinical models [136] and in humans [137]. Mechanistic studies in rodents have attributed depressive-like behaviour to a reduced neuron density and long-term potentiation (LTP) deficits in the hippocampus, reduced BDNF, and reduced serotonin in several brain regions, including the PFC [138,139,140,141,142]. Despite the apparent evidence of prenatal pro-inflammatory cytokines in programming offspring behaviour, it is important to note that maternal inflammation and foetal or neonatal exposure to maternal inflammatory mediators do not necessarily equate to a similar inflammatory response in the offspring [134]. It is largely unknown how elevated maternal cytokines alter brain development, though it is possible that they more directly affect synaptic plasticity and pruning and neuronal function in offspring [143]. Given the broad range of behavioural outcomes in offspring associated with maternal immune activation, it seems unlikely that maternal inflammation is correlated with a specific disease. Rather, MIA may contribute to a wider spectrum of psychiatric illnesses, the penetrance of which may depend on other genetic and environmental factors. In humans, therefore, maternal infection and inflammation may act as a disease primer, increasing the risk of psychopathology manifesting later in life.

Obesity is an inflammatory condition. While different to an acute inflammatory episode, obesity is known to be associated with a prolonged increase in circulating pro-inflammatory cytokines, including IL-6 [144]. Pregnancy is normally associated with immunological changes, including enhanced pro-inflammatory responses to lipopolysaccharide (LPS) and viral challenges [145]. Obesity during gestation exacerbates inflammatory changes associated with pregnancy, which has implications for foetal development [146]. Maternal obesity during pregnancy in humans is associated with significant alterations to the placenta, including increased resident CD14+ and CD68+ macrophages, and an increase in the placental expression of several pro-inflammatory cytokines, including IL-1β, TNF, and IL-6 [147,148]. Placental inflammation due to high-fat-diet consumption has been demonstrated in non-human primates [149]. Significant endocrine changes in the placenta also occur in response to maternal inflammation, mediated by IL-6 [150]. Increased maternal plasma concentrations of IL-6 were also observed in obese pregnant women compared to lean pregnant women [147,151]. Increased levels of endothelial intercellular adhesion molecule 1 and reduced levels of the anti-inflammatory cytokine IL-10 have also been observed early in pregnancy in obese pregnant women compared to lean pregnant women [151]. Increased serum IL-6 has been replicated in rodent models of maternal obesity, with a concomitant reduction in placental vascularisation [152]. Additionally, it has been shown that maternal IL-6 may transfer across the placenta and into foetal circulation [153]. Gestational weight gain, as opposed to pre-pregnancy obesity, may also be important. One study identified that every kilogram of gestational weight gain was associated with a 3% increase in serum C-reactive protein (CRP) and serum amyloid A (SAA) levels independent of pre-pregnancy BMI [154], though other inflammatory markers were unaffected. These studies clearly indicate that obesity during gestation may impact foetal development via excessive pro-inflammatory immune mediators.

The link between brain, behaviour, and immunity is evident in sickness behaviour. This is a robust evolutionary response that arises upon the administration of a peripheral inflammatory stimulus such as an endotoxin in both rodents [155] and humans [156]. Early life infection may also result in persistent changes to the brain and behaviour. For example, a longitudinal study in humans found an association between early-life (<14 years of age) viral CNS infection and the risk of schizophrenia development in adulthood [157]. Other studies have discovered a similar association after a bacterial infection [158,159]. Interestingly, the link between depression and immunity may be bidirectional, as depression at the baseline has been shown to be prospectively associated with an increased risk of hospitalisation due to infection in the UK Biobank [160]. Strong epidemiological evidence has found that the risk of developing any common mental disorder was increased with childhood infection that required hospitalisation or other treatment [161]. While the trigger in this case may be caused by an infectious agent, peripheral inflammation is known to lead to the de novo synthesis of cytokines in the brain [162]. Therefore, adaptive CNS changes may occur via immune-mediated inflammatory pathways within the CNS. Chronic, stress-induced inflammation is thought to increase the risk of depression and other mood disorders via similar inflammatory mechanisms [163]. Psychological stress, a key contributor to depression and other psychiatric conditions [164], alters the neuroimmune system, particularly the induction of a pro-inflammatory state with cytokine signalling initiated by activated microglia [165,166,167,168]. In humans, inflammatory biomarkers are associated with depression and depressive symptomology [169,170], and attenuating this immune activation may be a prerequisite for antidepressant treatment efficacy [171]. Rodent models of early-life stress have connected the development of depressive-like behaviours with an increased inflammatory tone that persists into adulthood [172]. In this way, mood and depression may have an inflammatory origin that is conditioned early in development.

Microglia and astrocytes have been shown to play an important role in many aspects of neurodevelopment. For example, they participate in the genesis and pruning of synapses, regulation of apoptosis, and vascularisation of the CNS [173,174]. The intimate relationship between microglia and neurons begins early in development, whereby microglia regulate and refine neural circuits, and relay peripheral information to the brain, including signals from the microbiota [175,176,177,178]. Cytokines, produced by glia in the CNS, are thought to promote forebrain development in utero [179], and, based on preclinical and in vitro models, appear to have broad, important roles as growth factors and in supporting gliogenesis in the CNS [180]. Of particular importance is IL-6, which plays a key role in both foetal brain development and in the association between maternal systemic inflammation and risk of psychiatric disorders in offspring [181]. Prenatal exposure to IL-6 was shown to cause deficits in spatial learning in adult offspring, with an accelerated hippocampal neurodegeneration and increased GFAP and NMDA NR1 subunit mRNA expression [182]. Additionally, maternal serum IL-6 levels during pregnancy correlated with functional and structural changes to the amygdala, as well as behavioural changes in 2-year-old human offspring [183]. Clearly, the biology of IL-6 and other cytokines during early neurodevelopment is complex, contributing to normal development and pathogenesis in a dose-dependent manner, via both direct and indirect mechanisms.

As in systemic infection, a maternal high-fat diet and obesity can impact the developing brain via inflammatory mechanisms. Increased circulating IL-6 and other pro-inflammatory mediators is associated with obesity during pregnancy in rodents [17] and humans [147,151,184,185,186,187,188,189,190,191], though the extent of elevation compared to lean women likely fluctuates over the course of pregnancy and postpartum [146,188,189]. This obesity-induced activation of the maternal peripheral immune system has been shown in rodents to affect the offspring brain and behaviour in a manner resembling the consequences of maternal or early-life infection. One of the first demonstrations of this was by Bilbo and Tsang (2010) [10]. In offspring exposed to maternal high-fat diet-induced obesity, increased hippocampal IL-1β protein, but not gene expression, and microglial activation were found in adulthood compared to those born to dams fed a low-fat diet. The offspring of dams fed the HFD also showed increased anxiety-like behaviour in the EPM. The study suggests that offspring microglia may be primed or sensitised to inflammatory responses in utero or early postnatal life due to maternal obesity, which persists into adulthood. Similar findings have been reported at weaning age, P21 [18]. Pro-inflammatory microglia that secrete more cytokines during this developmental period may contribute to the neuropathology of affective disorder associated with persistent central inflammation [192].

Microglial activation has been discovered post-mortem in depressed victims of suicide. Increased *CCL2* expression was identified in the anterior cingulate cortex relative to non-psychiatric controls. An increase in the number of perivascular macrophages was also identified, suggesting the further recruitment of circulating monocytes into the anterior cingulate cortex [193]. Conversely, antidepressant treatment has been shown to limit microglial activation associated with depressive-like behaviours in mice [194]. Indeed, research on the relationship between microglial activation and mood disorders remains almost exclusively preclinical [195]. Many animal studies have provided direct evidence for microglial activation in the foetal and adult brain after elevated maternal systemic inflammation, while other studies report synaptic and neurotransmitter deficits in the absence of microglial activation [196]. Microglia, therefore, may play a role in the effect of a maternal high-fat diet on brain development and behaviour. Creating an inflammatory environment at such early periods during development, with or without direct changes to foetal microglia, appears to have significant, enduring consequences to the brain and behaviour.

### 3.4. Breast Milk Composition

Unlike human milk substitutes (formula), human milk is highly dynamic in composition, varying within and between mothers, over time, and across populations [197]. For example, colostrum, the first milk produced after birth, is low in volume but rich in immunologic (antibodies for example) and developmental components (growth factors for example) when compared to milk produced in the later stages of lactation [198]. The composition of milk changes over the course of lactation, with human milk being considered fully mature by four to six weeks postpartum. Despite the variation in composition, all human milk throughout development contains essential nutrients and bioactive factors needed for infant health and development, such as a largely complete macronutrient profile, as well as essential micronutrients, immunoglobulins, prebiotics, and probiotics [197].

Indeed, while evidence for the importance of human breast milk on developing offspring metabolism, immunity, and commensal gut microbe colonisation is considerable [8], the connection to psychopathology is only being made relatively recently [199]. Breast milk is a source of live bacteria for the infant, possibly originating from the maternal gut (the putative entero-mammary pathway) [200], or the infant oral cavity (retrograde pathway) [201]. The human milk microbiome changes over the course of lactation, with possible implications for the infant microbiome. For example, the microbiome isolated from the colostrum is thought to be more similar to the maternal gut microbiome, whereas milk at 1 and 6 months of lactation aligns more with the infant oral cavity [202]. As a result, changes to the maternal gut microbiome have direct consequences for the seeding of microbes in offspring through breast milk at the earliest stages of life, and, through this, may affect offspring neurodevelopment [203]. In addition to essential macronutrients and micronutrients, breast milk also contains prebiotic human milk oligosaccharides (HMOs), which not only help to proliferate but also shape optimal gut bacteria populations in offspring [204] and prevent infection from pathogenic microorganisms [205]. The HMO composition in human milk can vary according to the maternal genotype [206], as well as the stage of lactation [207]. For example, the total HMOs were identified to be 17.7 g/L in colostrum, 13.3 g/L in transitional milk, and 11.3 g/L in mature milk [207]. However, the abundance of individual HMOs across the lactational stage also exhibits distinct patterns according to the lactational stage and maternal genotype [208]. This points to the overall importance of providing human breast milk as opposed to formula milk for the long-term metabolic health of developing offspring [209].

Breast milk may also program adverse outcomes in offspring if the maternal diet is lacking certain micronutrients, or, more likely in the UK, inducing maternal obesity. Maternal BMI influences the overall breast milk composition [201]. Mothers with a BMI >25 kg/m^2^ have higher levels of insulin in the breast milk and, despite an overall reduction in the fatty acid content, show an increase in pro-inflammatory fatty acids such as linoleic acid, an omega-6 fatty acid, and palmitic acid [210]. The microbiota composition of breast milk from obese mothers is less diverse [202] and contains lower levels of *Bifidobacterium* than lean mothers [211]. This is significant as *Bifidobacterium longum* subsp. *Infantis* present in human milk is predicted to metabolise many HMOs present in human milk [212], from which SCFAs such as acetate and butyrate are produced. Moreover, an elevated BMI has been suggested to modify the immunological composition of breast milk, with high levels of IL-6 [211], leptin [213,214], and other pro-inflammatory mediators [215] reported. Lastly, the maternal BMI shows a positive association with Caesarian delivery as opposed to natural birth [216], a negative association with the rate and time to onset of breastfeeding [217,218], and a reduction in the manual expression of milk compared to the use of a breast pump [218]. These factors likely also contribute to changes in the infant microbiome. For example, indirect (breast pump) vs. direct (skin–skin contact) breastfeeding has been associated with a reduction in the diversity of the milk microbiome, as well as an enrichment in potentially pathogenic bacteria and a reduction in *Bifidobacteria* spp. [201]. It also suggests that the retrograde pathway of milk microbiota colonization from the infant oral cavity is an important factor in the benefits of manual milk expression.

It is also worth noting that the introduction of solid food into the infants’ diet also has a dramatic effect on the gut microbiota composition [219,220]. *Bacteroidetes* dramatically increase in abundance, which may serve to assist in the metabolism of more complex polysaccharides [219]. Conflicting evidence exists as to whether the cessation of breastfeeding or introduction of solid foods is the predominant mediator of microbiome maturation in infants, though it is likely that both factors play a contributary role [220]. Importantly, evidence exists to support a continued beneficial role of breast milk (as oppose to milk substitutes) during the introduction of solid food, whereby human milk suppressed the solid-food-mediated increase in *Firmicutes* in the infant gut [220]. An increased *Firmicutes/Bacteroidetes* ratio is often observed in obese individuals [221,222]. Additionally, the maternal dietary practices associated with maternal obesity and healthy weight likely lead to altered postnatal feeding practices in the offspring. This is an important consideration in the impact of solid food on the infant gut microbiota and how the food composition may also differ between mothers who are obese or of healthy weight [223].

Clinical studies investigating the role of breastfeeding in the vertical transmission of psychopathology to the offspring face significant socioeconomic confounders. Initial studies on breastfeeding and its relationship with neurodevelopment focused on cognitive outcomes, whereby breastfeeding was generally associated with predicting greater academic achievements compared to formula feeding [224,225]. In preterm infants, breastmilk was also found to increase the rate of regional gray matter development when compared with formula-fed infants [226]. This observational study is the first to suggest that human milk exerts a direct and distinct effect on the brain structural development in humans. However, in these clinical studies, it is not possible to randomise mothers and their newborns to formula and breastfeeding groups, and therefore confounders may exist. Additionally, because both obesity [227] and perinatal mental illness [228,229] are associated with socioeconomic inequality in the UK and globally, observational studies in humans have significant limitations [230]. When trying to observe the effect of maternal obesity and depression, independently or together against a control cohort, on the breast milk composition and its effect on childhood psychopathology development, obese/depressed mothers are more likely to switch to formula milk sooner [231], have a lower income, have difficult social environments, and have reduced social support, and the young offspring are more likely to receive lower education standards. These factors independently increase the risk of adverse childhood neurodevelopment [232].

Studies in mice have provided direct evidence for maternal obesity during lactation, having the ability to program metabolic inflammation [233] and precocious pubertal development [234] in the offspring. The latter finding was shown to be reversible with maternal microbial reconstitution during lactation, pointing to the role of the altered vertical transmission of microbes and associated microbial metabolites during lactation as a mediator of adverse metabolic programming in offspring [234]. In our laboratory, we reported a reduction in milk butyrate levels in obese dams compared to lean dams, in line with perturbations to the maternal microbiome following HFD-induced obesity [12]. This observation is supported by work in humans, which has shown that a diet high in fat (predominately soybean oil) reduces the gut microbial diversity and total SCFA levels in a controlled isocaloric study relative to a low-fat diet [235]. Because the dietary fibre intake was matched between the HFD and control diet, it is possible that the reduced *Bifidobacteria* and butyrate levels observed in HFD-fed dams is due to the much lower carbohydrate level compared to the control diet. In humans, a low-carbohydrate diet is associated with reduced faecal butyrate and butyrate-producing bacteria levels [236]. While the level of *Bifidobacterium* spp. was not quantitated in the milk of the dams, the observation of reduced faecal *Bifidobacterium* spp. may have mediated a decrease in the milk levels of *Bifidobacterium* spp. and butyrate. Certain species of Bifidobacterium present in human milk are thought to metabolise milk oligosaccharides into SCFAs such as butyrate [212]. In line with this, maternal probiotic supplementation during gestation increases the levels of SCFAs and lactate in milk at postnatal day 4 [12]. Rodent [114] and human [237] studies have demonstrated the important neuromodulatory roles of gut and maternal-derived metabolites at similar developmental stages. Other bioactive molecules contained in breast milk, such as miRNAs, are differentially abundant in the milk of lean and obese dams [238]. These miRNAs, contained in extracellular vesicles, have been shown to survive a simulation of gastric digestion in vitro, though their functions in milk remain largely unknown [239].

Concerning the altered composition of breast milk in humans, few studies have related this to maternal psychopathology. Based on a recent review [199] of maternal psychopathology and altered breast milk composition, perinatal depression does not appear to significantly affect the micronutrient content, though omega-3 polyunsaturated fatty acids (PUFAs) may be reduced in depression. Docosahexaenoic acid (DHA) is an omega-3 PUFA crucial for neurodevelopment [240,241], and reduced levels have been linked to MDD in humans. Perinatally anxious and depressed mothers may also have increased secreted IgA in breast milk, which has been suggested to be a protective mechanism against other depressive-associated alterations in the mother that might otherwise be transmitted in the milk [199]. Overall, there is a narrow scope of literature with a heterogeneous mix of human observational studies addressing how maternal breast milk components may contribute to offspring brain and behavioural development. Given that early-life offspring are dependent on breast milk for nutritional nourishment and immune mediators while also being a critical source of commensal microbes, there is scant research on how different maternal nutritional and psychological states affect the breast milk composition. Furthermore, there is preliminary evidence to suggest that maternal psychopathologies and obesity independently alter the breast milk composition to the detriment of the offspring—likely by microbial and immune mechanisms. There remains a need for preclinical studies to investigate in more detail how depressive and anxious behaviours, in the presence of obesity, influence the bioactive content of milk throughout the nursing period. The metagenomic and transcriptomic content of milk in relation to the maternal diet and behaviour are also lacking in current research.

### 3.5. Neurotransmitter Systems

The developing neurotransmitter systems of offspring exposed to maternal obesity have been suggested to be influenced by immune and microbial factors, which have been addressed in the previous sections. Moreover, the opportunity to ameliorate substantial changes to neurotransmission may exist only in infancy. Alterations to serotonin availability during foetal development alters the normal formation of brain circuitry, resulting in enduring changes to adult anxiety-like behaviours [242,243]. An early study investigating the role of commensal gut flora on serotonergic neurodevelopment found that recolonisation post-weaning was unable to normalise the increased levels of serotonin and its primary metabolite 5-HIAA in germ-free mice [244]. While the complete lack of microbes in early life has been suggested to increase serotonin levels, a maternal high-fat diet consumption may conversely suppress the offspring serotonergic system [11].

Maternal systemic inflammation during pregnancy may contribute to initial serotonin deficits in offspring. This has been suggested to occur by promoting placental kynurenine metabolism from tryptophan through the upregulation of indoleamine-2,3-dioxygenase (*IDO1*) expression, a rate-limiting enzyme in the kynurenine metabolic pathway. This depletes the foetal tryptophan availability in the foetus and limits the central serotonin availability and production. Preclinically, this has been shown to occur in the murine placenta following prenatal infection and immune activation [245]. Neonatal rabbits exposed to endotoxins in utero also show a reduced tryptophan metabolism to serotonin in the cortex and paraventricular region as measured by α[^11^C]methyl-l-tryptophan using positron emission tomography in vivo imaging [246]. Similarly, cortical and hippocampal serotonin levels as measured by an enzyme-linked immunosorbent assay (ELISA) were reduced in the newborn rabbit brain exposed to prenatal endotoxin compared to the PBS control. Toll-like receptor 4 (TLR4) is critically involved in inflammatory responses to endotoxins [247] and is a gatekeeper for the downstream induction of pro-inflammatory pathways, but, during gestation, can also respond to sterile pro-inflammatory signalling [248,249]. TLR4 activation initiates a host of cytokine responses and pro-inflammatory effector molecules via the nuclear translocation of nuclear factor kappa B (NFkB). The upregulation of maternal TLR4 is a risk for pre-term birth and foetal inflammatory response syndrome, characterised by the systemic activation of the foetal immune system [249]. In humans, TLR4 expression was shown to be upregulated 9–10-fold in the placentas of obese women compared to lean women. The expression of pro-inflammatory cytokines was also significantly greater [250]. This may have implications for excessive pro-inflammatory signalling in the foetus, though more studies assessing the relationship between an increased maternal/placental TLR4 expression and foetal inflammation, with outcomes assessing neurotransmission and behaviour, are required.

The placenta is an essential exogenous source of serotonin for the developing foetus. This was first shown by Bonnin et al. (2011), whereby maternal tryptophan was metabolised to serotonin in the placenta (shown in both mice and humans) before entering foetal circulation and allowing for normal forebrain development [251]. Placental-derived serotonin is crucial for developing forebrain neurocircuitry, and its disruption by inflammation could be a mechanism by which prenatal maternal inflammation increases the risk of subsequent mental illness. Maternal immune activation in mice has been shown to alter placental serotonin production. Surprisingly, placental serotonin production from tryptophan increased with maternal inflammation [252]. Despite no increase in the expression of pro-inflammatory cytokines in the placenta or the foetal brain, placental *IDO1* expression was shown to increase 4-fold 24 h after maternal immune activation with poly(I:C), and placental *tryptophan hydroxylase 1 (TPH1)* increased 2.5-fold. After another 24 h, *TPH1* expression was significantly reduced compared to saline controls. Remarkably, this window of increased *TPH1* expression rapidly increased the placental serotonin output, as well as foetal serotonin specifically in the forebrain (at gestational day 14). Kynurenine was also increased in the forebrain. This increase in foetal forebrain serotonin levels blunted serotonergic axon outgrowth [252]. While no behavioural experiments were conducted, the authors suggested that this phenomenon is maladaptive and may mediate, in part, the effect of maternal inflammation on increasing the risk of offspring anxiety and depressive-like behaviours in the offspring.

Preclinical studies have also investigated the effect of maternal-diet-induced obesity on serotonergic perturbations in offspring, both at the juvenile age and in adulthood. Sullivan et al. (2010) showed that maternal high-fat-diet consumption increased foetal tryptophan hydroxylase 2 (*TPH2*) expression in the rostral dorsal raphe region of NHP macaques, as well as the upregulation of the inhibitory 5-HT_1A_ receptor [11]. *TPH2* is expressed in serotonergic neurons and is critical to the central production of serotonin. An increase in foetal serotonin here may have also caused the subsequent reduced serotonin levels in the cerebrospinal fluid (CSF) at juvenile age (4 months) reported in the study by Sullivan et al. (2010), with concomitant increased anxiety-like behaviour in female offspring [11]. This study demonstrates that similar perturbations to serotonergic neurodevelopment may occur as a result of maternal metabolic inflammation, akin to maternal immune activation. A more recent study [19] extended these findings to adult NHPs (11 months), with reduced serotonin levels, an altered *TPH2* expression, and increased anxiety-like behaviours persisting despite being weaned onto a regular diet. These changes were exacerbated if the NHPs exposed to a high-fat maternal diet were also weaned onto the same diet. Overall, preclinical studies suggest that an impaired development of the offspring serotonin system characterised by altered serotonergic activity may increase the risk of offspring behavioural problems with enduring effects.

Based on the postulated action of the antidepressant selective serotonin reuptake inhibitor (SSRI) agents, it is widely regarded that serotonergic neurotransmission plays a role in depression and anxiety in humans. In cohorts vulnerable to depression, reduced dietary tryptophan and subsequent serotonin deficits can induce temporary depressive symptoms [253]. In terms of TPH2 functionality in humans, polymorphisms in *TPH2* have also been associated with depression and anxiety [254]. Perturbations to serotonin neurotransmission are associated with anxiety and depressive disorders in humans [255]. Reduced serotonin neurotransmission may underly depressive behaviour, with an increase in neurotransmission thought to be a requirement for successful treatment with the selective serotonin reuptake inhibitor (SSRI) citalopram [256]. This may depend on a reduced sensitivity over time to the inhibitory 5-HT_1A_ receptor, and hippocampal neurogenesis [255]. Perhaps in some individuals in which SSRIs are effective, this is correcting for serotonergic deficits that began in neurodevelopment. While maternal obesity has been associated with precocious puberty in females [257], there is a lack of understanding as to how this also translates to an altered brain function. Preclinical studies should further investigate the role of maternal pregravid obesity on brain development, and how this may translate to adversely affect the development of normal emotional behaviours in humans.

## 4. Perturbed Metabolism in Offspring Exposed to Maternal Obesity

The metabolic effects of chronic maternal HFD feeding are known to propagate to the offspring. For example, maternal obesity results in hepatic steatosis and an impaired insulin sensitivity in rodent offspring [258,259,260] and dyslipidaemia in humans [261]. While alterations in peripheral lipid metabolites are relatively well documented, few studies have investigated the brain metabolome of offspring exposed to maternal obesity [20,262]. In one study, the effect of a maternal diet (HFD vs. control) was explored in male F1 six-month-old offspring (six per group) in the liver, hypothalamus, and olfactory bulb by mass spectrometry (MS). While the maternal diet had a strong effect on programming hepatic amino acid metabolism in the offspring, researchers found no lasting effects of the maternal diet on brain metabolites in the two brain regions [20]. By contrast, Zhu et al. (2018) demonstrated that the offspring of obese dams can be discriminated with 100% accuracy from the offspring of lean dams based on brain PFC metabolites alone, also using MS techniques [262]. Male F1 offspring (6–7-months-old, n = eight per group) from either obese LEPRdb/+ or lean WT dams were compared, whereby the maternal mice heterozygous for leptin receptor deficiency displayed hyperphagia and a 33% weight gain compared to WT mice prior to conception. Both saturated and unsaturated fatty acids were found to have accumulated in the PFC of the LEPRdb/+ offspring compared to the WT offspring. However, because the genotype of the offspring also differed between the two groups in the same way as the F0 generation, it is unclear whether maternal obesity or offspring genotype was responsible for the observed increase in PFC lipid levels. Considering this conflicting evidence, it is important for future studies to determine how maternal obesity alters the offspring brain metabolome whilst considering possible effects of offspring sex and the longevity of such changes.

In the juvenile offspring of HFD-fed dams, 3-hydroxybutyric acid was found to be significantly elevated in the brain compared to the offspring of lean dams [12]. 3-hydroxybutyric acid is thought to mediate neural stress responses; for example, due to fasting, ketogenic diets, or exercise [263]. The increase in brain 3-hydroxybutyric acid in the juvenile offspring may be a direct result of increased fatty acid oxidation from the increased abundance of lipids present in the milk of obese dams [264]. This is supported by the fact that, in the aforementioned study, 3-hydroxybutyric acid was also significantly elevated in the plasma of the juvenile offspring of obese dams relative to the offspring of CD dams [12]. Furthermore, a concomitant decrease in faecal glucose and succinate metabolites in HFD offspring compared to CD offspring suggests that the juvenile offspring exposure to maternal obesity induced a shift in the gut microbiota ecology [12,265].

Lastly, we have reported maternal obesity to reduce brain and liver creatine levels in the offspring [12]. A systematic review [266] collated data from studies employing in vivo brain 1H magnetic resonance spectroscopy in MDD patients and found that creatine and glutamate were significantly downregulated in MDD. A low dietary creatine intake is a clinical risk factor for depression [267], and creatine supplementation has shown promise as an adjunct supplement for MDD [268]. A promising theory for the pathophysiology of synaptic dysfunction in psychiatric disorders is that plastic changes are constrained by brain energy deficits [269]. This has been supported by preclinical [270] and clinical evidence [271] implicating reduced creatine in depression and reduced creatine kinase flux in bipolar disorder, respectively. The results from our metabolomic study support accumulating evidence connecting maternal obesity during pregnancy to long-term changes in central metabolism and behaviour [272].

## 5. Overview of Maternal Influences on the Offspring and Looking beyond the First Filial Generation

**Figure 2:**
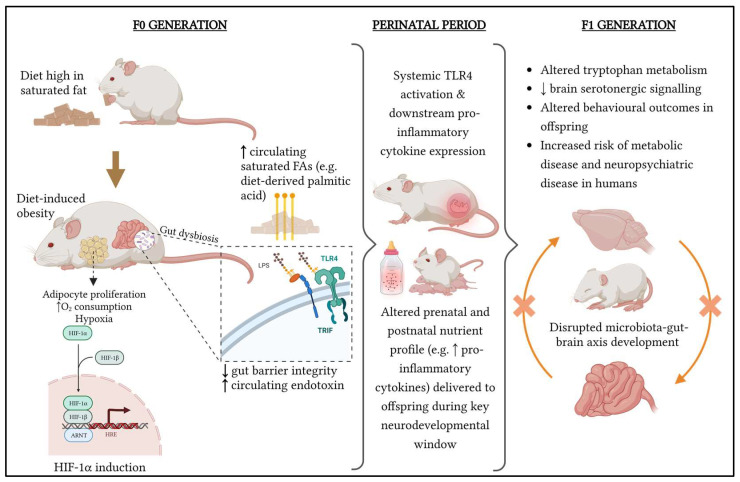
Mechanistic overview of the convergent mechanisms by which maternal-diet-induced obesity and microbiome dysbiosis induce toll-like receptor 4 (TLR4) activation, increasing maternal inflammation during the perinatal period and disrupting the development of the gut–brain axis in the offspring.

Factors associated with maternal pregravid obesity should not be considered in isolation when investigating their effects on the offspring brain and behaviour (Figure 2). Multiple perturbations coalesce to alter the normal course of neurodevelopment in offspring. For example, TLR4 activation links maternal obesity with increased immune activation via both an altered microbiota composition (endotoxin mediated inflammation), adipocyte proliferation (hypoxia and HIF-1 mediated inflammation [273]), and by diet, whereby saturated fatty acids can also activate TLR4 [274,275,276] (Figure 2). A state of chronic low-grade inflammation, exacerbated by normal immunological changes that occur during gestation, is likely to have an impact on the development of normal brain circuitry, particularly through alterations in the availability of serotonin during key neurodevelopmental windows. Sex differences may also occur, with females perhaps being more vulnerable to the effects of maternal obesity on developing depressive behaviour.

Given that depression and stress are also linked to excessive GWG [277], the effect of maternal obesity on offspring neurobiology and behaviour, coupled with adverse metabolic outcomes, may contribute to a perpetual cycle of poor maternal health across generations. The impact of the maternal obesity of a generation across subsequent generations was explored in a recent study [21]. The F0 consumption of a diet consisting of 60% of kilocalories from fat for 14 weeks primed anxiety-like behaviours as assessed in the light–dark box (LDB) and elevated plus maze in female F2 (granddaughter) offspring. This was suggested to be due to heritable changes in the HPA-axis functionality as assessed by gene expression analysis, specifically the mineralocorticoid receptor (MR) in the hippocampus. Metabolic changes were also associated with the F0 diet [21]. This study emphasises the importance of understanding sexual dimorphism in intergenerational behavioural studies and highlights the need for pharmacological and/or lifestyle interventions to aid in reducing the intergenerational matrilineal transmission of vulnerability to mental illness.

## 6. Translational Perspectives and Conclusions

In humans, lifestyle modifications are key to reducing the risk of maternal complications during pregnancy and supporting healthy offspring development. Current UK guidelines emphasise the importance of preventative strategies, whereby a healthy body mass index is achieved prior to conception [278,279]. Nevertheless, the prevalence of maternal obesity in the UK doubled between 1989 and 2007 from 7.6% to 15.6% [280], and may now be as high as 25% [3]. As pharmacotherapy, including weight-loss therapy, during pregnancy raises safety concerns, optimising maternal nutrition during pregnancy and breastfeeding is a crucial aspect of pregnancy care [279]. An established example of this is the relationship between maternal obesity, the increased risk of neural tube defects in the offspring, and folic acid supplementation. Obese women of childbearing age are known to have reduced serum folate levels, even when adjusting for folate intake through diet or supplements [281,282,283]. The underlying mechanisms for this may relate to differences in the gut microbiota composition, chronic inflammation, insulin resistance, and epigenetic factors between obese and lean women [284]. As a result, guidelines for folate supplementation from one month preconception through the first trimester of pregnancy have changed from a “one-dose fits all approach” to a higher dosage (5 mg daily as opposed to 0.4 mg daily) in obese women.

This tailored approach to maternal nutrition could, in the future, be extended to target the gut (and milk) microbiota to reduce the risk of disease in the offspring, including mood disorders. Preliminary clinical evidence exists to support this idea. A study of >20,000 mother–child pairs revealed that the children of mothers who followed an “unhealthy diet” high in processed foods during pregnancy were more likely to demonstrate externalising behavioural problems at 5 years of age compared to the children of mothers who followed a healthy diet [285]. This finding was independent of social deprivation, but it was unclear if it was independent of a maternal BMI. More recently, it has been demonstrated in 213 mother–infant pairs that the maternal prenatal gut microbiota composition was predictive of internalising behaviour in children at 2 years of age [286]. This study is the first to report a longitudinal association between maternal microbiota and offspring behaviour in humans, which previously had been reported in animal studies. Surprisingly, this effect was not mediated by infant gut microbiota. The maternal microbiota diversity was not predictive of the child microbiota diversity at 6 months of age, and there was no relationship between the child gut microbiota composition at 6 months of age and behaviour at 2 years of age. A mediation analysis suggested that, while there was no overall association between the maternal diet quality and offspring behaviour, the maternal diet modified the maternal gut microbiota diversity, which likely indirectly influenced offspring behaviour outcomes [286]. A genus-level analysis of maternal microbiota revealed that the levels of butyrate-producing bacteria (*Lachnospiraceae* and *Ruminococcaceae*) were lower in mothers with children who exhibited internalising behavioural problems. No direct association between maternal serum or faecal acetate, propionate, or butyrate levels and offspring behaviour was found, but samples acquired for SCFA analysis were taken at a different point in gestation (28 weeks) to the faecal microbiota sampling (36 weeks). In summary, this recent prospective human study supports current preclinical evidence that the maternal microbiota is associated with offspring development and behaviour. Continuing to assess child and adolescent behaviour in the future in this longitudinal fashion will be important in assessing the longevity of this association. Future studies in this area should aim to perform a metagenomic analysis of the gut microbiome to retrieve information about the collective microbiota function as opposed to taxonomy only. As microbiota metabolism is thought to be more conserved between individuals than taxa [287], this approach may lead to more precise findings and shed more light on the metabolic changes occurring during offspring development in utero.

Microbiota modification by probiotic treatment or other methods may also aid directly in preventing excessive gestational weight gain and improving the maternal metabolic response to a dietary intervention. Turnbaugh et al. (2006) introduced the concept of an “obese-type” gut microbiome, the metabolic potential of which may account for why some people find it difficult to lose weight [288]. By extension, gut microbes associated with obesity could be targets for therapeutic intervention. In another study, faecal metagenomic analysis distinguished individuals who were either susceptible or resistant to a dietary weight-loss intervention [289]. Differentially expressed microbial genes included an increase in starch-degrading amylases in those resistant to weight loss, which was independent of the baseline BMI. Taken together, gut microbiota have the potential to modify energy harvest in humans. While there is some evidence to suggest that probiotic treatment can help with weight loss in humans [290], it is unclear whether probiotics are more or less effective than increasing the daily fibre intake, which has also been shown to result in clinically-significant weight loss in humans [291].

Overall, dietary changes as recommended by a dietitian or other health professional are likely to be an appropriate first-line measure for women seeking to improve their metabolic health prior to, during, and after pregnancy. However, based on the results in this thesis and in line with current literature, a direct modification of the maternal microbiota may also be appropriate to mitigate the risk of metabolic and/or mood disorders in the offspring. For example, in cases of a low adherence to lifestyle interventions, antibiotic usage, or where dietary changes are not feasible during pregnancy, personalised probiotic treatment may be warranted. Clinical research in this area should be encouraged to assess the efficacy of such an intervention.

## Figures and Tables

**Table 1:** Overview of preclinical studies investigating mechanisms of maternal-diet-induced obesity on the offspring brain and behaviour.

Species (Strain)	Maternal Diet	Intervention Period, Length	Offspring Behavioural Outcomes	Offspring Brain Outcomes	Author, Year
Mouse (CD-1)	HFD (60% kcal fat) vs. CD (10% kcal fat)	Prenatal + postnatal, 12 weeks (including 6 weeks pre-gestation)	↑ anxiety OFT PND21 and PND112; ↑ passive stress coping FST PND21 and PND112; ↓ social interaction PND56	↓ brain creatine and brain glutamate PND21; ↑ PFC *ZIF-268* mRNA PND21; ↓ PFC *SYP* mRNA PND112	Radford-Smith et al., 2022 [12]
Rat (Sprague Dawley)	HFD (60% kcal sat. fat) vs. trans fat (60% kcal trans-fat) vs. CD (10% kcal fat)	Prenatal + postnatal, 10 weeks (including 4 weeks pre-gestation)	↑ anxiety EPM (males only), ↑ memory performance MWM in adulthood (3 months age)	↑ hippocampal microglial activation (*CD11b, TLR4*) at PND1, ↑ IL-1B protein at PND20, ↑ hippocampal microglial activation and reactivity in adulthood (3 months age)	Bilbo and Tsang, 2010 [10]
Japanese macaques	HFD (32% kcal fat) vs. CD (13% kcal fat)	Up to 4 years (including prenatally)	↑ anxiety female juvenile offspring (PND130)	↑ *TPH2, 5-HT_1A_R* in dorsal raphe in foetuses;	Sullivan et al., 2010 [11]
Rat (Wistar)	HFD (40% kcal fat) vs. CD (12% kcal fat)	Gestation at lactation (until PND21)	↓ anxiety in OFT, ↑ social interaction in HFD adult offspring experiencing maternal separation in early life compared to CD adult offspring	Prevents neurobiological effects (↓ *BDNF, 5-HT_1A_R, CRH*) of maternal separation stress in juvenile offspring (PND11) and anxiety-induced cFOS and corticosterone in adulthood (8 months).	Rincel et al., 2016 [13]
Mouse (C57Bl/6)	HFD (45% kcal fat) vs. CD (10% kcal fat)	6 weeks (including gestation)	Pups exposed to HFD in utero altered maternal care behaviour (reduced time spent on nest) independent of maternal diet (cross-fostering experiment)	-	Baptissart et al., 2018 [14]
Rat (Long Evans)	HFD (60% kcal fat) vs. chow diet (13.5% kcal fat)	~6 weeks (including 3 weeks pre-gestation)	Pup isolation test: HFD pups vocalised less at PND7, more at PND13	↑ *CRH,* ↓ *NR3C1* in paraventricular nucleus at PND7, ↑ *GAD1,* ↓ *NR3C1* in ventral hippocampus at PND13	Abuaish et al., 2018 [15]
Mouse (C57Bl/6)	HFD (60% kcal fat) vs. chow diet (13.4% kcal fat)	Prenatal + postnatal, 14 weeks (including 8 weeks pre-gestation)	↓ social interaction (3-chamber social interaction test), ↑ anxiety (OFT and marble burying) 7–12 week offspring	↓ hypothalamic oxytocin in HFD offspring; ↓ social interaction-induced dopaminergic activity in ventral tegmental area	Buffington et al., 2016 [16]
Mouse (C57Bl/6)	HFD (45% kcal fat) vs. CD (10% kcal fat)	Variable pre-gestation until significant weight gain occurred	-	↓ neuropeptide Y innervation of paraventricular nucleus in foetal offspring	Sanders et al., 2014 [17]
Mouse (C57Bl/6)	Chronic mild stress + Western diet (40% kcal fat) vs. no stress CD (10% kcal fat)	Prenatal + postnatal, 11 weeks (including 5 weeks pre-gestation)	-	↑ hippocampal microglial activation (↑ CD11b+ cells, ↑ *AIF1*, *TLR9* mRNA and protein); ↓ neuronal cell density (NeuN+ cells) in hippocampus	Cohen et al., 2016 [18]
Japanese macaques	HFD (36.6% kcal fat) vs. CD (14.7% kcal fat)	Mothers had been consuming HFD for 1.2–8.5 years at birth of offspring	↑ anxiety and stereotypic behaviours in offspring (11 months age)	↓ *TPH2* mRNA in dorsal raphe; ↓ serotonin immunoreactivity prefrontal cortex (13 months age)	Thompson et al., 2017 [19]
Mouse (C57Bl/6)	HFD (60% kcal fat) vs. CD (10% kcal fat) vs. weight loss (HFD → CD)	Prenatal + postnatal, 5.5 months (including 4 months pre-gestation)	-	No significant effect of maternal diet on offspring hypothalamus or olfactory bulb metabolite levels	Safi-Stibler et al., 2020 [20]
Rat (Sprague Dawley)	Grandmaternal HFD (60% kcal fat) vs. CD (10% kcal fat)	Prenatal + postnatal, 14 weeks (including 8 weeks pre-gestation)	↑ anxiety in female F2 offspring in EPM	↑ hippocampal *CRH-R2* in male HFD F2 offspring; ↑ *NR3C2* in female HFD F2 offspring	Winther et al., 2019 [21]

HFD, high fat diet; CD, control diet; PND, postnatal day; OFT, open field test; FST, forced swim test; EPM, elevated plus maze; MWM, Morris water maze; PFC, prefrontal cortex; *ZIF-268* encodes zinc finger protein 268 (early growth response protein 1); *SYP* encodes synaptophysin; *CD11b* encodes integrin alpha M; *TLR4* encodes toll-like receptor 4; IL, interleukin; *TPH2* encodes tryptophan hydroxylase; *5-HT_1A_R* encodes the serotonin 1A receptor; *BDNF* encodes brain-derived neurotrophic factor; *CRH* encodes corticotropin releasing hormone; *NR3C1* encodes the glucocorticoid receptor; *AIF1* encodes allograft inflammatory factor 1; *TLR9* encodes toll-like receptor 9; *CRH-R2* encodes corticotropin-releasing hormone receptor 2; *NR3C2* encodes the mineralcorticoid receptor.
